# Hormonal Contraceptive Use and Musculoskeletal Injury Risk in Female Athletes: A Prospective Cohort Study

**DOI:** 10.1177/19417381261459590

**Published:** 2026-07-23

**Authors:** Natasha Trentacosta, Giselle Kaneda, Natasha Schimmoeller, Dave T. Huang, Jasmine Galloway, Marilee Fisher, Chloe Castaneda, Christian Blough, Thomas Kremen, Caroline Jefferies, Dmitriy Sheyn, Melodie F. Metzger

**Affiliations:** Department of Orthopedics Cedars-Sinai Medical Center (CSMC), Los Angeles, California; Orthopaedic Stem Cell Research Laboratory at CSMC, Los Angeles, California, Board of Governors Regenerative Medicine Institute at CSMC, Los Angeles, California, and Department of Biomedical Sciences, CSMC, Los Angeles, California; Department of Obstetrics and Gynecology CSMC, Los Angeles, California; Department of Orthopedics Cedars-Sinai Medical Center (CSMC), Los Angeles, California, and Orthopedic Biomechanics Laboratory CSMC, Los Angeles, California; Cedars-Sinai Kerlan Jobe Institute, Los Angeles, California; University of California, Los Angeles, California; Department of Orthopedics Cedars-Sinai Medical Center (CSMC), Los Angeles, California Department of Orthopedics Cedars-Sinai Medical Center (CSMC), Los Angeles, California and Orthopedic Biomechanics Laboratory CSMC, Los Angeles, California; Department of Orthopedics Cedars-Sinai Medical Center (CSMC), Los Angeles, California; University of California, Los Angeles, California; Department of Biomedical Sciences, CSMC, Los Angeles, California, Kao Autoimmunity Institute, CSMC, Los Angeles, California, and Department of Medicine, Division of Rheumatology, CSMC, Los Angeles, California; Department of Orthopedics Cedars-Sinai Medical Center (CSMC), Los Angeles, California, Orthopaedic Stem Cell Research Laboratory at CSMC, Los Angeles, California, Board of Governors Regenerative Medicine Institute at CSMC, Los Angeles, California, Department of Biomedical Sciences, CSMC, Los Angeles, California, and Department of Surgery, CSMC, Los Angeles, California; Department of Orthopedics Cedars-Sinai Medical Center (CSMC), Los Angeles, California, and Orthopedic Biomechanics Laboratory CSMC, Los Angeles, California

**Keywords:** ACL, birth control, female athlete, hormonal contraceptives, ligament, sex hormones

## Abstract

**Background:**

Female reproductive hormones are attributed to the higher rate of musculoskeletal (MSK) injuries among female athletes compared with male athletes.

**Purpose:**

To evaluate association between exposure to hormonal contraceptives (HCs), joint laxity, and number of injuries in female athletes over a 1-year period.

**Study Design:**

Cohort study.

**Level of Evidence:**

Level 3.

**Methods:**

Professional and Division I collegiate female athletes were recruited into 2 groups based on HC status (HC vs non-HC). Serum relaxin, estrogen and progesterone levels, knee laxity, generalized hypermobility, and lower extremity kinematics during a single-legged jump were examined during preseason in the luteal phase of each athlete’s menstrual cycle. Injuries were tracked for 1 year after testing.

**Results:**

Circulating levels of progesterone (non-HC, 38.9 ± 15.0 pg/mg vs HC, 28.6 ± 11.4 pg/mg; *P* < 0.01), estrogen (non-HC, 2.80 ± 3.0 pg/mg vs HC, 2.0 ± 3.0 pg/mg; *P* = 0.01), and relaxin (non-HC, 0.26 ± 0.08 pg/mg vs HC, 0.22 ± 0.03 pg/mg; *P* = 0.04) were lower in HC (n = 32) than in non-HC (n = 40) athletes during the luteal phase of the cycle. Non-HC athletes demonstrated significantly greater hip flexion at initial contact (non-HC, 29.42°± 7.64° vs HC, 25.25°± 7.13°; *P* = 0.02), and greater knee adduction (valgus) at maximum knee flexion (non-HC, –1.90°± 3.25° vs HC, –0.25° ± 3.40°; *P* = 0.02) during a single-legged drop. Average (±SD) injury count for non-HC athletes was 0.71 (±1.3) compared with 0.35 (±0.70) in the HC group (*P* = 0.25). Injury count was correlated significantly to circulating levels of relaxin (*r* = 0.32; *P* < 0.01).

**Conclusion:**

A potential relationship between increased circulating relaxin levels and the risk of injury was established. Athletes taking HCs demonstrated significantly reduced peak knee adduction angles when landing from a jump.

**Clinical Relevance:**

The results advance knowledge of relationships between specific hormones and injury risk, and the potential role of HCs on kinematic patterns when landing from a jump. Future research will determine HC formulations that may protect against MSK injury, and inform broader strategies to reduce sport-related injury risk in female athletes.

The dramatic increase in female participation in sports after Title IV passed in 1972 created a need to better understand the health and medical wellbeing of this growing athletic population. One of the first studies to analyze the susceptibility of female athletes to injury concluded that “well-trained” women sustained the same injuries at the same rate as men, and that any differences in frequency would likely adjust once “better coaches and trainers became available to women.”^24^ However, with time and data collected from the National Collegiate Athletic Association (NCAA), it became clear that female athletes playing pivoting sports had a significantly greater rate of noncontact ligament injuries, including anterior cruciate ligament (ACL) ruptures, compared with male athletes playing the same sport, year after year.^1,5,8,22,38^ Further research has confirmed this sex disparity in musculoskeletal (MSK) tissue exists among other active populations, including high school athletes and military personnel.^11,40,42,55,57^

While these sex-differences are widely accepted, the exact reason behind them is still not fully understood and is likely multifactorial.^20^ Higher rates of ACL tears among female athletes have been associated with several anatomical, biomechanical, and neuromuscular risk factors. Anatomically, women generally have a wider pelvis, greater quadriceps (Q) angle, and a smaller femoral notch, all of which have been associated with an increased likelihood of injury. Biomechanical and neuromuscular factors that further increase risk include greater joint laxity, increased dynamic knee valgus, quadraceps-dominant movement patterns, reduced core strength, and delayed neuromuscular control.^2,9,26,39,44,64^ At the tissue level, the female ACL is typically smaller, has lower mechanical strength, and reduced collagen fibril density.^23^ While these factors may contribute, substantial clinical and basic science evidence suggests that fluctuations in female reproductive hormones significantly alter the mechanical properties of the ACL but the underlying mechanism remains unclear.^15,18,32,33^

The monthly cycling of female reproductive hormones is driven by the hypothalamic-pituitary-ovarian (HPO) axis, which stimulates ovulation midcycle. After a mature egg is released from the ovary, the originating follicle transforms into the corpus luteum, which is a temporary structure that releases hormones to prepare the body for a possible pregnancy. One of the hormones released is relaxin, which allows the MSK system to adapt to a pregnancy should it occur.^13,41,54^ In women who do not conceive, this increase in relaxin may be associated with adverse effects, including a possible increased susceptibility to ACL rupture.^15,17,33^

Hormonal contraceptives (HCs) prevent pregnancy by providing a steady level of exogenous hormone that suppresses the HPO axis and thus inhibits ovulation. While previous studies have indicated HCs may play a protective role in reducing MSK injuries in female athletes, these are limited to case-control or retrospective analyses.^14,21,25,33,47,49,56^ Therefore, the purpose of this study was to prospectively evaluate hormonal regulation via HCs to changes in ligament function via joint stability and risk of injury in high-level female athletes.

## Methods

### Participant Recruitment

**Figure 1.**
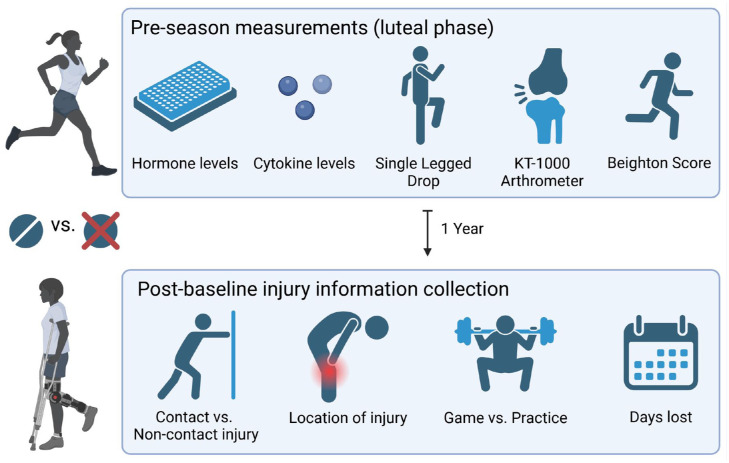
Schematic of study design comparing female athletes on or not on HCs. HC, hormonal contraceptive.

Institutional review board (IRB) approval and informed consent were obtained before initiating the study. Team doctors, coaching staff, and athletic trainers were contacted from surrounding NCAA Division I and professional-level athletic programs, specifically from sports involving pivoting maneuvers, including basketball, soccer, and volleyball. A meeting with each team was arranged during their respective preseason to distribute a 1-page flyer and provide a brief overview of the study. Athletes who were interested in participating completed a consent form and questionnaire. The questionnaire gathered basic demographics, medical and surgical history, the date of the first day of their last menstrual cycle, and information about birth control use, including the type, name, and brand, if applicable. Athletes were excluded if they had a history or current pregnancy or a history of previous bilateral ACL ruptures to reduce variability that might have been introduced with the procedure. After receiving completed forms, baseline data were collected in the training rooms during the luteal phase of each athlete’s menstrual cycle, calculated from the first day of their last period noted in their questionnaire. Baseline data included collection of a 5-ml blood sample, assessment of lower body kinematics, evaluation of general hypermobility, and measurement of knee laxity using the KT-1000 arthrometer (Figure 1).

### Ligament and Joint Laxity

Anterior-posterior (A-P) knee laxity was measured using the KT-1000 arthrometer by a single member of our research team. Measurements were obtained at 30 lbs of anterior force, with the device’s audible tone confirming that the target force had been reached; 3 trials were recorded on both the left and right knees and averaged. In addition, each athlete was guided through the 9-point Beighton score assessment to evaluate overall hypermobility. The intrarater intraclass correlation coefficient (ICC) for KT-1000 measurements have been reported as moderate to good (0.36-0.94) and Beighton score intrarater ICC is considered above moderate to excellent (0.71-0.98), supporting these measures for assessing knee laxity and joint hypermobility.^48,52^

### Kinematic Analysis

An Xsens Awinda Inertial Measurement Unit (IMU) system was used to capture lower body movements at 120 Hz using a multifloor configuration. A total of 7 IMUs were attached to the lower body: 1 on the pelvic bone, 1 on each thigh, 1 on each shank, and 1 on each foot. After attachment, the system was calibrated according to the manufacturer's recommendations to align sensor coordinate frames with body segments. Briefly, participants stood in the “I” pose, defined as a neutral position with the hips, knees, and ankles in 0 degrees of flexion, for 5 seconds. This was followed by 15 seconds of normal walking and then returning to the “I” pose for another 5 seconds. This system has been used widely in clinical and research settings and achieves orientation accuracy of up to 0.5° root mean square (RMS) for roll/pitch and 1.0° RMS for yaw.^3,12,46,62^

Once calibrated, participants performed a single-legged drop, a widely used tool that replicates high-risk biomechanics associated with noncontact, using their dominant leg from a 12-inch exercise stool. The dominant leg was identified by asking the participant, “Which leg do you usually use to kick a soccer ball or football?” Participants were then instructed on how to perform the single-legged drop: arms crossed in front of the chest, nondominant leg raised so that the thigh is parallel to the floor, and, finally, jumping off from the stool using their dominant leg, and remaining as stable as possible after landing. Each participant performed 5 single-legged drop trials. A prespecified rule required that, once landed, the foot remain in contact with the ground for ≥2 seconds for the trial to be considered successful; 5 successful trials were collected for each participant. Any trial with event-specific joint angles exceeding ±3 SD of that participant’s 5-trial mean was excluded during data postprocessing. Joint angles were then averaged across the 5 successful trials. To limit inter-rater variability, a single experienced biomechanist delivered a standardized script and demonstration, performed all IMU sensor placements, and used an identical processing workflow for all participants.

**Figure 2.**
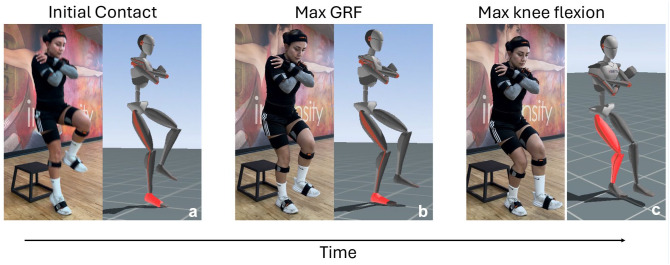
Timepoints of measurement for single-legged drop. (a) Initial contact, (b) maximum GRF, and (c) maximum knee flexion. GRF, ground reaction force.

Joint angles were processed using the ZYX rotation convention in MVN Analyze (v2022.0, Movella). A customized Python script was used to analyze 3 specific points in time to examine lower body joint angles: (1) the initial landing phase, identified by the first positive spike in vertical axis acceleration on the IMU attached to the landing foot, corresponding to initial ground contact; (2) the maximum landing force phase, marked by the second positive spike in vertical axis acceleration on the landing foot’s IMU, indicating peak force during landing; and (3) the moment of maximum knee flexion, determined by the knee joint angle (Figure 2).

### Serum/Plasma Processing

Blood was transported to the laboratory and processed within 24 hours of collection. Whole blood was centrifuged for 10 minutes at 1000*g* and the resulting serum/plasma aliquoted and frozen at –80°C for later use.

### Hormone Quantification

Total serum and plasma protein concentration were determined via Pierce BCA Protein Assay (1:5 dilution, Thermo Fisher). Blood progesterone (Enzo Life Sciences Inc) and relaxin (R&D Systems) levels were assessed using enzyme-linked immunosorbent assay (ELISA) on serum according to manufacturer protocol. Total estrogen levels were assessed using ELISA (Eagle Biosciences) on plasma according to manufacturer protocol. All hormone levels were normalized to total protein concentration.

### Cytokine Quantification

Previous literature has demonstrated inflammation significantly influences outcomes after ACL rupture, and it is well established that women exhibit heightened levels of immune response compared with men.^6,30,31^ However, to our knowledge, no study has examined the possible impact of baseline system inflammation on ACL injury risk. To address this, blood cytokine levels were quantified using a 13-plex Inflammation panel LEDGENDplex flow assay (Biolegend) detecting interleukin (IL)-1β, interferon (IFN)-α2, IFNγ, tumor necrosis factor (TNF)-α, monocyte chemoattractant protein (MCP)-1, IL-6, IL-8, IL-10, IL-12p70, IL-17A, IL-18, IL-23, and IL-33. Serum samples were diluted 1:2 in assay buffer and run according to manufacturer protocol. Fluorescent intensity was acquired using a Sony ID7000 Spectral Cell Analyzer (Sony). The resulting data were analyzed using the online LEGENDplex analysis software.

### Injury Tracking

Injury tracking for the female athletes was conducted through regular communication between study staff and the athletic trainers of each team. Study staff maintained monthly contact with the athletic trainers throughout the athletes’ competitive seasons. An injury tracking form was completed collaboratively by the athletic trainers and study staff to document each reported MSK injury, defined as a diagnosed injury beyond pain alone (strain, sprain, fracture, etc). Injuries were recorded regardless of whether they resulted in missed games or practices, although time loss was noted when available. The form included the date of the injury, anatomical location, whether the injury occurred during a game or practice, whether it was contact or noncontact, and the specific injury type. Injury data collection began at baseline and continued through the end of each athlete’s season. Baseline injury and surgical history were recorded separately; injuries captured during follow-up represented injuries occurring during the study period and were not limited to first-ever injuries. Reinjury status was not adjudicated uniformly and was not used as an exclusion criterion. In addition, we used this monthly check-in to ask whether the HC status had changed for any of the participants.

### Statistical Analysis

No human studies were available to estimate an effect size, so an a priori power analysis was conducted based on differences in ACL strength between rats on and not on oral contraceptive formulations suggesting approximately 36 participants per group would be needed to reach a power of 80% with significance set at 0.05 and a calculated effect size of 1 to 1.3.^32^ Continuous variables were assessed for normality using the Shapiro-Wilk test. For normally distributed variables, independent samples *t* tests were performed to compare differences between the non-HC and HC groups. Non-normally distributed variables were compared using Mann-Whitney *U* tests. Chi-squared tests were used to determine and evaluate differences in categorical variables. For multiple pairwise comparisons, significance thresholds were adjusted using the Bonferroni correction. Pearson’s correlation coefficients were calculated to assess the magnitude and direction of linear relationships between circulating hormone levels, biomechanical outcomes, and injury status and were interpreted using conventional thresholds (weak |r| < 0.3, moderate |r| = 0.3-0.5, and strong |r| > 0.5). Odds of injury were evaluated using bivariate logistical regression. A significance level of *P* < 0.05 was used for all analyses. Chi-squared tests and correlation analyses were performed using R Software (https://www.R-project.org/).

## Results

### Participant Demographics

A total of 72 women were recruited to participate in the study, n = 40 in the non-HC and n = 32 in the HC group (HC included IUD or oral contraceptives). The average age of the athletes was 21.54 ± 3.42 years old, and the average height was 174.24 ± 9.29 cm tall. There was no statistical difference in age between non-HC and HC athletes for either age (non-HC, 21.80 ± 3.6 years; HC, 21.22 ± 3.21 years; *P* = 0.76) or height (non-HC, 173.61 ± 9.07 cm; HC, 175.82 ± 8.23 cm; *P* = 0.28) between the 2 groups. Participants played basketball (n = 20; non-HC, n = 12; HC, n = 8), beach volleyball (n = 19, non-HC, n = 9; HC, n = 10), and soccer (n = 33; non-HC, n = 19; HC, n = 14). No statistically significant association was observed between sport played and HC use (*P* = 0.53).

### Hormone Quantification

**Figure 3.**
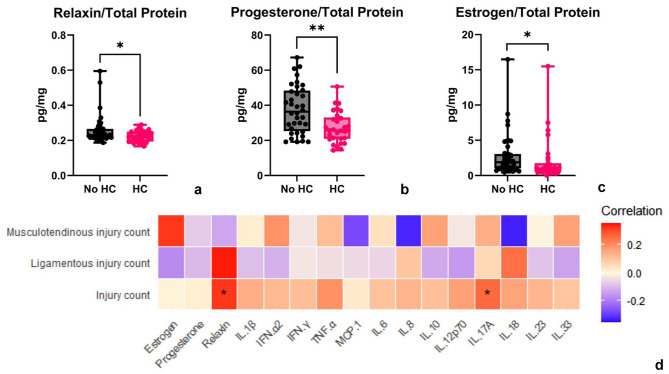
Quantification of blood (a) relaxin, (b) progesterone, and (c) estrogen levels at baseline. Blood relaxin, progesterone, and estrogen levels normalize to total protein count. (d) Correlation of hormone and cytokine measurements with injury count. *P ≤ 0.05, **P ≤ 0.01. HC, hormonal contraceptive; IFN, interferon; IL, interleukin; MCP, monocyte chemoattractant protein; TNF, tumor necrosis factor.

ELISA-based quantification of reproductive hormones revealed significant differences between HC and non-HC participants. Relaxin levels were significantly higher in non-HC compared with HC athletes (non-HC, 0.26 ± 0.08 pg/mg; HC, 0.22 ± 0.03 pg/mg; *P* = 0.04) (Figure 3a). Progesterone levels were significantly higher in non-HC than HC participants (non-HC, 38.9 ± 15.0 pg/mg; HC, 28.6 ± 11.4 pg/mg; *P* < 0.01) (Figure 3b). Similarly, estrogen levels were also significantly higher in non-HC than HC participants (non-HC, 2.80 ± 3.0 pg/mg; HC, 2.0 ± 3.0 pg/mg; *P* = 0.011) (Figure 3c).

### Injury Tracking

During the duration of the study, 24 athletes reported injuries for a total of 39 injuries. Of the 24 athletes reporting injuries, 16 were non-HC athletes, who received a total of 29 injuries, and 8 were HC athletes, who received a total of 10 injuries, which was not statistically different between groups (*P* = 0.25). As expected, the number of reported injuries corresponded to the number of participants in each sport, with soccer having the highest (n = 13), followed by basketball (n = 8), and beach volleyball (n = 3). Most injuries occurred during practice (n = 19) and games (n = 15), with the vast majority of injuries occurring in the joints of the lower extremities (90%), namely knees and ankles (knees = 6, ankles = 18). Of injuries reported, 27 were ligamentous, 10 were musculotendinous, and 2 were osseous.

Total injury count was correlated positively significantly to serum relaxin (*r* = 0.321 (moderate); *P* = 0.007) (Figure 3d). However, no significant correlations were observed between relaxin and ligamentous injuries (*r* = 0.341; *P* = 0.12) or musculotendinous injuries (*r* = –0.127; *P* = 0.572), when accounted for separately (Figure 3d). Estrogen (*r* = 0.01; *P* = 0.95) and progesterone (*r* = 0.017; *P* = 0.89) were not correlated significantly with injury count, ligamentous injury count, or musculotendinous injury count (Figure 3d).

### Ligament and Joint Laxity

**Figure 4.**
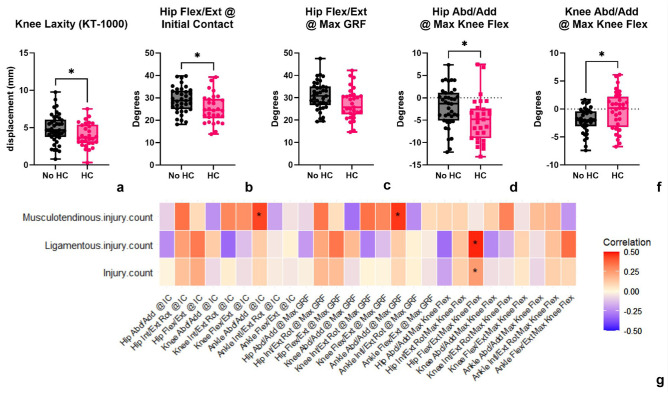
Significant differences in KT-1000 and hip, knee, and ankle kinematics from the single-legged drop. (a) KT-1000, (b) hip Flex/Ext at initial contact, (c) hip Flex/Ext at maximum GRF, (d) hip Ab/Ad at Max knee Flex, (e) and knee Ab/Ad at Max knee Flex. (f) Correlation of kinematic measurements with injury count. *P ≤ 0.05. Ab, abduction; Ad, adduction; Ext, extension; Flex, flexion; GRF, ground reaction force; HC, hormonal contraception; Max, maximum.

KT-1000 measurement for knee laxity showed significant differences between the non-HC and HC groups (non-HC, 4.84 ± 1.93 mm; HC, 3.95 ± 1.58 mm; *P* = 0.04) (Figure 4a). Beighton score showed no difference between non-HC and HC groups in either upper body (non-HC, 2.05 ± 1.89; HC, 1.69 ± 1.91; *P* = 0.34), lower body (non-HC, 0.52 ± 0.85; HC, 0.25 ± 0.67; *P* = 0.10), or collective score (non-HC, 3.17 ± 2.62; HC, 2.47 ± 2.36; *P* = 0.26). There were no significant correlations between KT-1000 or Beighton measures and any of the hormones investigated.

### Kinematic Analysis

Motion tracking analysis of the athletes during a single-legged drop identified several significant differences in lower limb kinematics between non-HC and HC athletes. Hip flexion was significantly higher in non-HC compared with HC athletes at initial contact (non-HC, 29.42 ± 7.64; HC, 25.25 ± 7.13; *P* = 0.02) (Figure 4b) and approached significance at the instant of highest landing force (non-HC, 30.87 ± 8.30; HC, 27.86 ± 7.69; *P* = 0.07) (Figure 4c). At the instant of maximum knee flexion, hip adduction was also found to be significantly higher in higher HC athletes compared with non-HC (non-HC, –2.01 ± 5.20; HC, –5.00 ± 6.85; *P* = 0.04) (Figure 4d), while knee adduction was found to be higher in non-HC athletes (non-HC, –1.90 ± 3.25; HC, –0.25 ± 3.40; *P* = 0.02) (Figure 4e).

Correlative analysis of single-legged drop kinematics with injuries demonstrated that hip flexion was correlated positively with both total injury count (*r* = 0.25 [weak]; *P* = 0.03) and ligamentous injury count (*r* = 0.48 [moderate]; *P* = 0.02) at instant of maximum knee flexion (Figure 4f). Likewise, hip flexion and progesterone levels were correlated positively and significantly at all measurement points (initial contact: *r* = 0.264 [weak]; *P* = 0.03; peak landing force: *r* = 0.315; *P* = 0.009; maximum knee flexion: *r* = 0.278; *P* = 0.02). Estrogen was also correlated positively and significantly with hip adduction at peak landing force (*r* = 0.23; *P* = 0.05) and approached significance for hip flexion at the same time point (*r* = 0.23; *P* = 0.06). In addition, relaxin levels were correlated positively and significantly with hip flexion at initial contact (*r* = 0.235; *P* = 0.05). Musculotendinous injuries count was correlated positively with ankle adduction both at initial contact (*r* = 0.44; *P* = 0.04) and the instant of max landing force (*r* = 0.46; *P* = 0.03) (Figure 4f).

### Cytokine Quantification and Correlation Analysis

**Figure 5.**
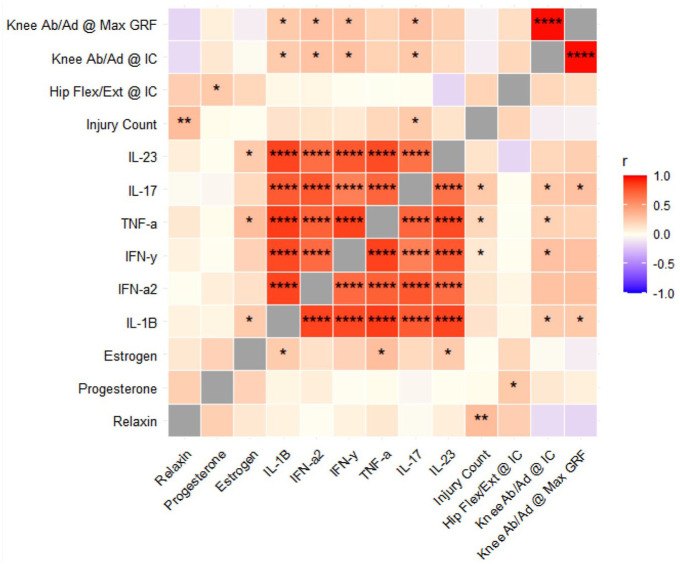
Correlations between selected measures. Ab, abduction; Ad, adduction; Ext, extension; Flex, flexion; GRF, ground reaction force; HC, hormonal contraceptive; IC, initial contact; IFN, interferon; IL, interleukin; Max, maximum; MCP, monocyte chemoattractant protein; TNF, tumor necrosis factor.

None of the 13 cytokines quantified were significantly different between the HC and non-HC groups. Only IL-17A was correlated positively and significantly with injury count (*r* = 0.258; *P* = 0.04); however, no significance was observed when looking separately at ligamentous injury count (*r* = 0.060; *P* = 0.78), or musculotendinous injury count (*r* = 0.140; *P* = 0.46). Correlation analysis between cytokines levels and relaxin or progesterone levels showed no significant correlation between any of the 13 measure cytokines. However, estrogen was found to be correlated positively and significantly with IL-1β (*r* = 0.25 [weak]; *P* = 0.05), TNF-α (*r* = 0.307 [moderate]; *P* = 0.01), and IL-23 (*r* = 0.247 [weak]; *P* = 0.05) (Figure 5).

### Risk Assessment

Although there was no significant association between HC use and risk of injury (relative risk [RR], 0.66; odds ratio [OR], 0.54; *P* = 0.24), risk assessed by hormone levels identified relaxin as a significant risk factor for injury. Specifically, athletes with serum relaxin levels in the fourth quartile (Q4) had approximately 2 times the risk of injury compared with those in the first through third quartiles (Q1-Q3), *P* = 0.02. Furthermore, athletes with serum relaxin levels in Q1 to Q3 reduced their odds of injury by 73% compared with those with relaxin levels in Q4 (OR, 0.27; 95% CI, 0.08-0.81; *P* = 0.02). When evaluating athletes in the lower half (Q1-Q2) compared with those in the upper half (Q3-Q4) of relaxin levels, athletes in Q1 to Q2 had a 56% lower risk of injury (RR, 0.44) and 70% lower odds of injury compared with Q3 to Q4 (OR, 0.30; 95% CI, 0.10-0.83; *P* = 0.03). While increased serum levels of estrogen and progesterone were associated with an increased risk and odds of injury, this was not significant.

## Discussion

The key finding of this study was that elevated serum relaxin was a significant risk factor for injury among female athletes (Figure 3d). Athletes with serum relaxin levels in the highest quartile experienced approximately twice the risk of injury compared with those with lower levels. In addition, those in the lowest half of relaxin concentrations had a 56% to 70% reduction in odds of injury. While no significant association was observed between HC use and injury risk, non-HC athletes had higher levels of serum relaxin (Figure 3a) and demonstrated greater knee adduction (Figure 4), which is a known risk factor for ACL rupture.^8,27^ We also report significant correlation of estrogen levels with some serum inflammatory cytokines, suggesting a regulatory role in inflammation that could be linked to injuries (Figure 5). Our results underscore the effect of female reproductive hormones on MSK function and suggest that modifying endogenous levels of hormones, potentially through contraceptive use, may reduce injury susceptibility in female athletes, particularly for ligamentous injuries.

Numerous studies have investigated the direct and indirect effects of cycling female sex hormones on MSK tissue and function.^10,28,29,45,50,58^ Wojtys et al^58^ analyzed 28 female athletes with ACL injuries and reported a significantly higher incidence during the ovulatory phase, with fewer injuries occurring during the follicular phase. Similarly, several studies have documented increased knee joint laxity during the ovulatory phase, coinciding with elevated estrogen levels compared with the luteal and follicular phases.^29,45,50,58^ Similarly, we observed no correlation between passive knee laxity (via KT-1000) and estrogen measured during the luteal phase. However, serum levels of estrogen, progesterone, relaxin, and knee laxity were all reduced significantly in women taking HCs during the luteal phase, suggesting exogenous hormones may modulate knee laxity through several mechanisms throughout the menstrual cycle.

Our data corroborate previous literature linking relaxin with injury susceptibility in female athletes. In 2003, Dragoo et al^16^ collected remnant ACL tissue from patients undergoing ACL reconstruction and found relaxin receptors were present on female, but not male, ACL tissue. In addition, female athletes with elevated serum relaxin levels, particularly during the luteal phase, have been associated with increased risk for ACL injury.^15^ In animal models, exogenous administration of relaxin has been shown to increase connective tissue laxity and reduced load to failure.^17,61^

Relaxin is a small peptide in the insulin-like superfamily that effects ligament remodeling directly by promoting activation of collagenase, matrix metalloproteinases (MMPs), and plasminogen activator.^13^ Of the 7 known relaxin family peptides, relaxin 1 and 2, in particular, are known to be involved in connective tissue regulation. These peptides bind to relaxin receptors 1 and 2, initiating intracellular cascades that increase MMP-1, -2, -3, -9, and -13, which degrade extracellular matrix proteins, including collagens. Thus, while relaxin plays a crucial role in preparing the body for labor and delivery by loosening connective tissue, our study suggests it also heightens a women’s vulnerability to serious sports injuries through a similar mechanism.

Although estrogen levels were elevated in non-HC athletes during the luteal phase of their cycle, it was not correlated with injury incidence, suggesting the role of estrogen during this phase is more subtle. Estrogen receptors were first identified on the ACL by Liu et al^36^ in 1996, and, while the mechanism is still not fully understood, studies demonstrate that estrogen has an effect on connective tissue remodeling and integrity. Estrogen directly alters ligament laxity through inhibition of fibroblast proliferation and a reduction of procollagen type I synthesis. It also plays an indirect role through enzymatic inhibition and transcriptional regulation of lysyl oxidase (LOX) - an enzyme essential for covalent cross-linking of collagen and elastin fibers.^35,63^

Interestingly, our results showed significant positive correlations between estrogen and several pro-inflammatory cytokines, specifically IL-1β, TNF-α, and IL-23. This aligns with previous research that demonstrates steroid hormones, including estrogen, effect cytokines by either upregulating or downregulating their expression, depending on the tissue type.^43,51^ IL-1β and TNF-α were also correlated with risk of injury in female athletes. These cytokines amplify inflammation by upregulating MMP and downregulating LOX expression, leading to tissue breakdown, hindered healing, and progressive joint degeneration.^37,60,65^ IL-23, which was also correlated with estrogen, is known primarily for its role in promoting Th17 cell differentiation and sustaining chronic inflammation in autoimmune diseases.^34^ Our results also showed that IL-17a, which functions downstream of IL-23 in the critical IL-17/IL-23 signaling axis, was correlated significantly with injury incidence.^34,53^ This is interesting, given that blood cytokine and hormone levels were collected at baseline, before injury, indicating that a pre-existing pro-inflammatory or immune-primed state, potentially influenced by estrogen, may predispose female athletes to ligament injury and impact healing trajectories negatively.

While we were unable to specifically measure the mechanical properties of participants MSK tissues in this in vivo study, several biomechanical measures that were recorded during the single-legged drop indicate decreased ligament function via increased joint laxity in women not taking HCs. Specifically, knee adduction (often referred to as valgus collapse) and hip flexion were significantly greater in non-HC athletes. While increased joint laxity may contribute to altered landing mechanics, differences in knee adduction and hip flexion likely reflect a combination of ligamentous properties and neuromuscular control (i.e., force closure and dynamic stability provided by muscle and tendon). Hip flexion was correlated to increased levels of progesterone and relaxin and was associated with an increased risk of injury. This is consistent with previous literature that links increases in female sex hormones, and these specific kinematic changes (knee valgus rotation and exaggerated hip flexion) to an increased risk of ACL injury.^4,7,8^

Our study had several limitations that should be considered. First, the specific type of hormonal contraceptive was not controlled as it would have limited participation. As a result, the dose, combination, and delivery method of estrogen and progestin (progesterone) varied among participants, which likely resulted in varying effects on the MSK system in the HC group. Indeed, a previous study examining different progestin-to-estrogen ratios in rats found that contraceptive formulations with higher progestin-to-estrogen ratios provided greater protective effect than those with lower ratios.^32^ Future studies focused on a specific formula of combined oral contraceptive pills with larger cohorts and more selective inclusion criteria are needed to help establish optimal formulations. Another limitation of this study was the reliance of participant questionnaire to estimate the luteal phase of each participant’s menstrual cycle and the collection of baseline measurements. Errors in recall and reporting inaccuracies may have compromised estimation of participant luteal phases and baseline data collection. In addition, strenuous exercise commonly associated with female athletes can result in irregular menstrual cycles, further complicating accurate determination of the luteal phase. Ideally, menstrual cycles should be based on hormonal profiles verified via blood analysis or urinary kits, with baseline venipunctures collected at the same time of day, which unfortunately was beyond the scope of our budget.^19^ We also did not collect information on the length of time users were on HCs. Our inability to capture greater detail on each athlete’s menstrual cycle and hormonal profile increases our between participant variability, which may have had implications on our findings, although, even without these details, there seems to be a relationship between relaxin and injury that is worth exploring further with a more optimized methodology. Furthermore, the highly significant difference in progesterone helps confirm the 2 groups of athletes due to its central role in ovulation and predictable suppression by HCs.^59^ Finally, some of our outcomes, particularly the correlations, are likely underpowered and we therefore caution against any conclusive statements while emphasizing more work on the female athlete is needed.

In conclusion, data collected in the present study establishes a potential relationship between increased circulating relaxin levels and the risk of injury. In addition, athletes taking HC demonstrated significantly reduced peak knee adduction angles when landing from a jump. Future research is necessary to determine the specific molecular mechanisms, as well as which HC formulations best prevent injuries and implement strategies that reduce the risk of sport-related injuries in young female athletes.

## Supplemental Material

sj-docx-1-sph-10.1177_19417381261459590 – Supplemental material for Hormonal Contraceptive Use and Musculoskeletal Injury Risk in Female Athletes: A Prospective Cohort Study Supplemental material, sj-docx-1-sph-10.1177_19417381261459590 for Hormonal Contraceptive Use and Musculoskeletal Injury Risk in Female Athletes: A Prospective Cohort Study by Natasha Trentacosta, Giselle Kaneda, Natasha Schimmoeller, Dave T. Huang, Jasmine Galloway, Marilee Fisher, Chloe Castaneda, Christian Blough, Thomas Kremen, Caroline Jefferies, Dmitriy Sheyn and Melodie F. Metzger in Sports Health

sj-docx-2-sph-10.1177_19417381261459590 – Supplemental material for Hormonal Contraceptive Use and Musculoskeletal Injury Risk in Female Athletes: A Prospective Cohort Study Supplemental material, sj-docx-2-sph-10.1177_19417381261459590 for Hormonal Contraceptive Use and Musculoskeletal Injury Risk in Female Athletes: A Prospective Cohort Study by Natasha Trentacosta, Giselle Kaneda, Natasha Schimmoeller, Dave T. Huang, Jasmine Galloway, Marilee Fisher, Chloe Castaneda, Christian Blough, Thomas Kremen, Caroline Jefferies, Dmitriy Sheyn and Melodie F. Metzger in Sports Health

sj-docx-3-sph-10.1177_19417381261459590 – Supplemental material for Hormonal Contraceptive Use and Musculoskeletal Injury Risk in Female Athletes: A Prospective Cohort Study Supplemental material, sj-docx-3-sph-10.1177_19417381261459590 for Hormonal Contraceptive Use and Musculoskeletal Injury Risk in Female Athletes: A Prospective Cohort Study by Natasha Trentacosta, Giselle Kaneda, Natasha Schimmoeller, Dave T. Huang, Jasmine Galloway, Marilee Fisher, Chloe Castaneda, Christian Blough, Thomas Kremen, Caroline Jefferies, Dmitriy Sheyn and Melodie F. Metzger in Sports Health
