# Supplementary material for: Hormonal Contraceptive Use and Musculoskeletal Injury Risk in Female Athletes: A Prospective Cohort Study
Source: Sports Health. 2026 Jul 23:19417381261459590. Online ahead of print. doi: 10.1177/19417381261459590 (PMC13400710; doi:10.1177/19417381261459590)
Supplement: sj-docx-1-sph-10.1177_19417381261459590 – Supplemental material for Hormonal Contraceptive Use and Musculoskeletal Injury Risk in Female Athletes: A Prospective Cohort Study [file sj-docx-1-sph-10.1177_19417381261459590.docx]

**Table S1.** Hormonal contraceptives (HC)used by study participants. N = number of subjects, mg = milligram, IUD = intrauterine device.

| **Hormonal Contraceptive (HC) Type** | **Patient Reported HC Name** | **Number of Athletes (N=32)** | **Daily ethinyl estradiol dose (mg)** | **Progestin type** | **Progestin dose (mg)** |
| --- | --- | --- | --- | --- | --- |
| IUD | Mirena | 3 | 0 | Levonorgestrel | 52 |
|  | Kyleena | 3 | 0 | Levonorgestrel | 19 |
|  | Unspecified homonal IUD | 2 | 0 | Levonorgestrel | 13.5-52 |
| Implant | Nexplanon | 1 | 0 | Etonogestrel | 68 |
| Ring | NuvaRing | 1 | 0.015 | Etonogestrel | 11.7 |
| Pill | Viorele | 1 | 0.02, 0.01 | Desogestrel | 0.15 |
|  | Isibloom | 1 | 0.03 | Desogestrel | 0.15 |
|  | Yaz, Loryna | 3 | 0.02 | Drospirenone | 3 |
|  | Yasmin | 2 | 0.03 | Drospirenone | 3 |
|  | Portia | 1 | 0.03 | Levonorgestrel | 0.15 |
|  | Blisovi FE 1/20, Junel 1/20, Loestrin | 4 | 0.02 | Norethindrone acetate | 1 |
|  | Ortho Tri-cyclen Lo | 1 | 0.025 | Norgestimate | 0.18, 0.215, 0.25 |
|  | Tri-Sprintec | 1 | 0.035 | Norgestimate (triphasic) | 0.18, 0.215, 0.25 |
|  | Ortho-cyclen, Sprintec | 3 | 0.035 | Norgestimate (monophasic) | 0.25 |
|  | Low-ogesterol | 1 | 0.03 | Norgestrel | 0.3 |
|  | Unspecified homonal pill | 3 | Unknown | Unknown |  |
|  | Progestin only pill | 1 | 0 | Norethindrone acetate | 0.35 |
