# Supplementary material for: Hormonal Contraceptive Use and Musculoskeletal Injury Risk in Female Athletes: A Prospective Cohort Study
Source: Sports Health. 2026 Jul 23:19417381261459590. Online ahead of print. doi: 10.1177/19417381261459590 (PMC13400710; doi:10.1177/19417381261459590)
Supplement: sj-docx-2-sph-10.1177_19417381261459590 – Supplemental material for Hormonal Contraceptive Use and Musculoskeletal Injury Risk in Female Athletes: A Prospective Cohort Study [file sj-docx-2-sph-10.1177_19417381261459590.docx]

**Table S2.** Reported energy deficiencies or menstrual dysfunction among participants on (HC) and not on (non-HC) hormonal contraceptives.

| **Condition** | **HC** | **Non-HC** |
| --- | --- | --- |
| Menorrhagia | 2 | 0 |
| Ovarian cysts | 1 | 0 |
| Eating disorder | 0 | 3 |
| Oligomenorrhea | 0 | 1 |
