# Supplementary material for: Hormonal Contraceptive Use and Musculoskeletal Injury Risk in Female Athletes: A Prospective Cohort Study
Source: Sports Health. 2026 Jul 23:19417381261459590. Online ahead of print. doi: 10.1177/19417381261459590 (PMC13400710; doi:10.1177/19417381261459590)
Supplement: sj-docx-3-sph-10.1177_19417381261459590 – Supplemental material for Hormonal Contraceptive Use and Musculoskeletal Injury Risk in Female Athletes: A Prospective Cohort Study [file sj-docx-3-sph-10.1177_19417381261459590.docx]

**Table S3.** Compiled list of injuries for participants on (HC) and not on (non-HC) hormonal contraceptives.

| **Injury** | **Non-HC** | **HC** |
| --- | --- | --- |
| ACL tear only | 1 | 0 |
| Meniscus tear only | 3 | 0 |
| ACL tear with meniscus tear | 1 | 1 |
| MCL sprain | 1 | 0 |
| Adductor strain | 1 | 1 |
| Ankle sprain | 13 | 3 |
| Foot sprain | 1 | 0 |
| Hamstring strain | 1 | 3 |
| Hip strain | 1 | 0 |
| Knee hyperextension | 1 | 0 |
| Plnatar fascia rupture | 1 | 0 |
| Re-reuptured plantar fascia | 1 | 0 |
| Thumb fracture | 2 | 0 |
| Thumb sprain | 1 | 0 |
| Posterior tibialis sprain | 0 | 1 |
| Sternoclavicular joint sprain | 0 | 1 |
